# Redetermination of Dy_3_Ni from single-crystal X-ray data

**DOI:** 10.1107/S1600536813028717

**Published:** 2013-10-26

**Authors:** Volodymyr Levytskyy, Volodymyr Babizhetskyy, Bohdan Kotur, Volodymyr Smetana

**Affiliations:** a Department of Inorganic Chemistry, Ivan Franko National University of Lviv, Kyryla & Mefodiya Street 6, 79005 Lviv, Ukraine; b 344 Spedding Hall, Ames Laboratory, Ames, IA 50011-3020, USA

## Abstract

The classification of the title compound, tridysprosium nickel, into the Fe_3_C (or Al_3_Ni) structure type has been deduced from powder X-ray diffraction data with lattice parameters reported in a previous study [Lemaire & Paccard (1967 ▶). *Bull. Soc. Fr. Mineral. Cristallogr.* **40**, 311–315]. The current re-investigation of Dy_3_Ni based on single-crystal X-ray data revealed atomic positional parameters and anisotropic displacement parameters with high precision. The asymmetric unit consists of two Dy and one Ni atoms. One Dy atom has site symmetry .*m*. (Wyckoff position 4*c*) and is surrounded by twelve Dy and three Ni atoms. The other Dy atom (site symmetry 1, 8*d*) has eleven Dy and three Ni atoms as neighbours, forming a distorted Frank–Kasper polyhedron. The coordination polyhedron of the Ni atom (.*m*., 4*c*) is a tricapped trigonal prism formed by nine Dy atoms.

## Related literature

For a previous crystallographic investigation of the title compound, see: Lemaire & Paccard (1967 ▶). For the Fe_3_C structure, see: Hendricks (1930 ▶), and for the Al_3_Ni structure, see: Bradley & Taylor (1937 ▶). For the Dy–Ni phase diagram, see: Zheng & Wang (1982 ▶). For magnetic properties of Dy_3_Ni, see: Talik *et al.* (1996 ▶), and for magnetic properties of Dy_3_Co, see: Baranov *et al.* (1995 ▶). For isotypic compounds, see: Tsvyashchenko (1986 ▶); Romaka *et al.* (2011 ▶); Buschow & van der Goot (1969 ▶); Givord & Lemaire (1971 ▶). For structure refinements of other compounds in the Dy–Ni system, see: Levytskyy *et al.* (2012*a* ▶,*b* ▶).

## Experimental

### 

#### Crystal data

Dy_3_Ni
*M* *_r_* = 546.21
Orthorhombic, 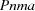

*a* = 6.863 (3) Å
*b* = 9.553 (3) Å
*c* = 6.302 (2) Å
*V* = 413.2 (3) Å^3^
*Z* = 4
Mo *K*α radiation
μ = 57.86 mm^−1^
*T* = 293 K
0.14 × 0.11 × 0.10 mm

#### Data collection

Stoe IPDS II diffractometer
Absorption correction: numerical (*X-RED*; Stoe & Cie, 2009 ▶) *T*_min_ = 0.007, *T*_max_ = 0.026
973 measured reflections
582 independent reflections
447 reflections with *I* > 2σ(*I*)
*R*_int_ = 0.042

#### Refinement

*R*[*F* ^2^ > 2σ(*F* ^2^)] = 0.042
*wR*(*F* ^2^) = 0.052
*S* = 1.12
582 reflections
23 parameters
Δρ_max_ = 2.82 e Å^−3^
Δρ_min_ = −2.65 e Å^−3^

### 

Data collection: *X-AREA* (Stoe & Cie, 2009 ▶); cell refinement: *X-AREA*; data reduction: *X-AREA*; program(s) used to solve structure: *SIR2011* (Burla *et al.*, 2012 ▶); program(s) used to refine structure: *SHELXL2013* (Sheldrick, 2008 ▶) and *WinGX* (Farrugia, 2012 ▶); molecular graphics: *DIAMOND* (Brandenburg, 2006 ▶); software used to prepare material for publication: *publCIF* (Westrip, 2010 ▶).

## Supplementary Material

Crystal structure: contains datablock(s) I, New_Global_Publ_Block. DOI: 10.1107/S1600536813028717/wm2777sup1.cif

Structure factors: contains datablock(s) I. DOI: 10.1107/S1600536813028717/wm2777Isup2.hkl

Additional supplementary materials:  crystallographic information; 3D view; checkCIF report

## Figures and Tables

**Fig. 1.**
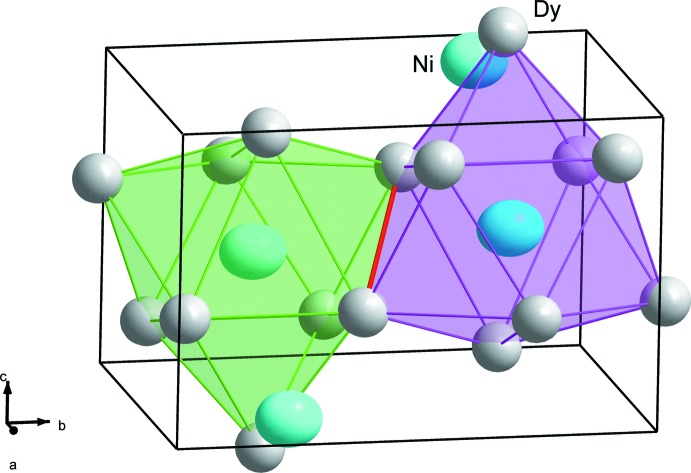
Perspective view of the crystal structure of Dy3Ni. The unit cell and coordination trigonal prisms for Ni atoms are emphasized. The stacking edge of these prisms is marked by red colour. Atoms are represented by their anisotropic displacement ellipsoids at the 99.9% probability level.

**Fig. 2.**
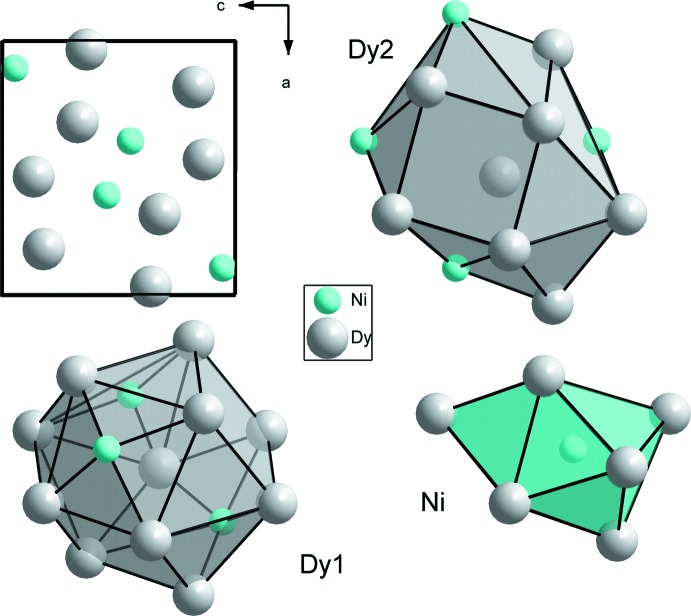
The ac projection of the unit cell and coordination polyhedra for all types of atoms in the Dy3Ni structure.

## supplementary crystallographic information

### 1. Comment

Lattice parameters for *RE*_3_Ni compounds with *RE* = Y, La, Pr, Nd, Sm, Gd–Tm, have been determined and their crystal structures reported to be isotypic with the Fe_3_C (or Al_3_Ni) type structure which includes also Dy_3_Ni (Lemaire & Paccard, 1967). According to the phase diagram of the Dy–Ni system (Zheng & Wang, 1982), Dy_3_Ni is stable below 1035 K and is formed by the peritectic reaction: Dy + *L*→ Dy_3_Ni.

Similar isotypic *RE*_3_Co compounds were also reported (Buschow & van der Goot, 1969) for *RE* = Y, La, Pr, Nd, Sm, Gd–Er. Lu_3_Co has been prepared by Givord & Lemaire (1971), Lu_3_Ni by Romaka *et al.* (2011). Tsvyashchenko (1986) synthesized Yb_3_Co and Yb_3_Ni at high pressure. According to Tsvyashchenko (1986), Yb_3_Co adopts the Fe_3_C type structure and Yb_3_Ni the Al_3_Ni structure type. On the other hand, Lemaire & Paccard (1967) claimed the *RE*_3_Ni compounds to have the same structure as the *RE*_3_Co compounds. To clarify the confusion with the assigned structure types, we have studied literature data for the Fe_3_C (Hendricks, 1930) and the Al_3_Ni (Bradley & Taylor, 1937) prototype structures, concluding that Al_3_Ni and Fe_3_C are isotypic. In accordance with the majority in literature, we will use the Fe_3_C structure type for classification as it has been reported earlier.

Recently, two binary compounds of the Dy–Ni system have been redetermined using single-crystal X-ray data (Levytskyy *et al.*, 2012**a*,b*). Here we present the results of the single-crystal X-ray analysis of Dy_3_Ni. Details of the crystal structure have not been investigated before, and only isotypism with the Fe_3_C was reported together with lattice parameters (Lemaire & Paccard, 1967).

The structure of Dy_3_Ni is characterized by formation of trigonal prisms of Dy atoms with Ni atom enclosed in the centre. A view of the crystal structure of Dy_3_Ni is shown in Fig. 1. The value of the displacement parameter *U*_22_ for the Ni atom displays a high anisotropy in the *b* direction which may have an influence on some physical properties of the compound. Magnetic properties of Dy_3_Ni were reported by Talik *et al.* (1996) and generally confirm this assumption which is also valid for the isotypic Dy_3_Co (Baranov *et al.*, 1995).

In Fig. 2 the *ac* projection of the unit cell and the coordination polyhedra for all atom types in Dy_3_Ni are shown. The coordination number for Dy1 (site symmetry .*m*., Wyckoff site 4 *c*) is 15 with bonding to 12 Dy and 3 Ni atoms. The coordination number for Dy2 (site symmetry 1, Wyckoff site 8 *d*) is 14, resulting in a distorted Frank–Kasper polyhedron defined by 11 Dy and 3 Ni atoms. The coordination number for Ni (site symmetry .*m*., Wyckoff site 4 *c*) is 9, resulting in a slightly distorted tricapped trigonal prism made up of 9 Dy atoms.

The analysis of interatomic distances shows a slight decrease of some Dy–Ni distances. This feature is in good agreement with the observed Ho–Co distances for previously reported Ho_3_Co (Buschow & van der Goot, 1969). The explanation of this fact may be deduced from an electronic band structure calculation.

### 2. Experimental

The sample was prepared from powdered commercially available pure elements: sublimed bulk pieces of dysprosium metal with a claimed purity of 99.99 at.% (Alfa Aesar, Johnson Matthey) and electrolytic nickel (99.99% pure) pieces (Aldrich). A mixture of the powders was compacted into a pellet. It was arc-melted under an argon atmosphere on a water-cooled copper hearth. The alloy button (~1 g) was turned over and remelted three times to improve homogeneity. Subsequently, the sample was annealed in an evacuated silica tube under an argon atmosphere for four weeks at 870 K. Shiny metallic gray plate-like crystals were isolated mechanically with a help of microscope by crushing the sample.

### 3. Refinement

The atomic positions found from the direct methods structure solution were in good agreement with those from the Fe_3_C structure type (Hendricks, 1930) and were used as starting point for the structure refinement. The highest Fourier difference peak of 2.82 e Å^-3^ is at (0.0340 0.75 0.1598) and 1.36 Å away from the Dy2 atom. The deepest hole of -2.65 e Å^-3^ is at (0.0358 0.25 0.0220) and 1.01 Å away from the Ni atom.

### Crystal data

**Table:**

Dy_3_Ni	*D*_x_ = 8.781 Mg m^−^^3^
*M**_r_* = 546.21	Mo *K*α radiation, λ = 0.71073 Å
Orthorhombic, *P**n**m**a*	Cell parameters from 2575 reflections
*a* = 6.863 (3) Å	θ = 3.7–29.5°
*b* = 9.553 (3) Å	µ = 57.86 mm^−^^1^
*c* = 6.302 (2) Å	*T* = 293 K
*V* = 413.2 (3) Å^3^	Block, metallic gray
*Z* = 4	0.14 × 0.11 × 0.10 mm
*F*(000) = 904	

### Data collection

**Table:**

Stoe IPDS II diffractometer	447 reflections with *I* > 2σ(*I*)
Radiation source: fine-focus sealed tube	*R*_int_ = 0.042
ω scans	θ_max_ = 29.6°, θ_min_ = 3.9°
Absorption correction: numerical (*X-RED*; Stoe & Cie, 2009)	*h* = 0→9
*T*_min_ = 0.007, *T*_max_ = 0.026	*k* = 0→12
973 measured reflections	*l* = −8→8
582 independent reflections	

### Refinement

**Table:**

Refinement on *F*^2^	Primary atom site location: structure-invariant direct methods
Least-squares matrix: full	Secondary atom site location: difference Fourier map
*R*[*F*^2^ > 2σ(*F*^2^)] = 0.042	*w* = 1/[σ^2^(*F*_o_^2^) + (0.0141*P*)^2^] where *P* = (*F*_o_^2^ + 2*F*_c_^2^)/3
*wR*(*F*^2^) = 0.052	(Δ/σ)_max_ < 0.001
*S* = 1.12	Δρ_max_ = 2.82 e Å^−^^3^
582 reflections	Δρ_min_ = −2.65 e Å^−^^3^
23 parameters	Extinction correction: *SHELXL2013* (Sheldrick, 2008), Fc^*^=kFc[1+0.001xFc^2^λ^3^/sin(2θ)]^-1/4^
0 restraints	Extinction coefficient: 0.00030 (10)

### Special details

**Table:**

Geometry. All e.s.d.'s (except the e.s.d. in the dihedral angle between two l.s. planes) are estimated using the full covariance matrix. The cell e.s.d.'s are taken into account individually in the estimation of e.s.d.'s in distances, angles and torsion angles; correlations between e.s.d.'s in cell parameters are only used when they are defined by crystal symmetry. An approximate (isotropic) treatment of cell e.s.d.'s is used for estimating e.s.d.'s involving l.s. planes.

### Fractional atomic coordinates and isotropic or equivalent isotropic displacement parameters (Å^2^)

**Table:**

	*x*	*y*	*z*	*U*_iso_*/*U*_eq_	
Dy1	0.17975 (10)	0.06439 (6)	0.17745 (9)	0.01479 (17)	
Dy2	0.03218 (14)	0.2500	0.63694 (13)	0.0147 (2)	
Ni	0.3917 (4)	0.2500	0.4477 (4)	0.0197 (5)	

### Atomic displacement parameters (Å^2^)

**Table:**

	*U*^11^	*U*^22^	*U*^33^	*U*^12^	*U*^13^	*U*^23^
Dy1	0.0150 (3)	0.0151 (3)	0.0143 (2)	0.0001 (3)	0.0003 (3)	−0.0004 (2)
Dy2	0.0158 (5)	0.0144 (4)	0.0138 (4)	0.000	0.0014 (3)	0.000
Ni	0.0131 (12)	0.0268 (14)	0.0192 (11)	0.000	0.0003 (10)	0.000

### Geometric parameters (Å, º)

**Table:**

Dy1—Ni^i^	2.770 (2)	Dy2—Dy1^xii^	3.5361 (12)
Dy1—Ni	2.856 (2)	Dy2—Dy1^vi^	3.5433 (12)
Dy1—Ni^ii^	3.3700 (15)	Dy2—Dy1^viii^	3.5944 (14)
Dy1—Dy1^iii^	3.5174 (11)	Dy2—Dy1^i^	3.5944 (14)
Dy1—Dy1^iv^	3.5174 (11)	Dy2—Dy1^xiii^	3.6047 (12)
Dy1—Dy2^v^	3.5361 (12)	Dy2—Dy1^iii^	3.6047 (12)
Dy1—Dy2	3.5433 (12)	Dy2—Dy2^xiv^	3.7156 (15)
Dy1—Dy1^vi^	3.5462 (16)	Dy2—Dy2^xi^	3.7156 (15)
Dy1—Dy1^vii^	3.5502 (15)	Ni—Dy1^x^	2.770 (2)
Dy1—Dy1^viii^	3.5512 (15)	Ni—Dy1^ix^	2.770 (2)
Dy1—Dy1^ix^	3.5512 (15)	Ni—Dy2^xiv^	2.790 (3)
Dy1—Dy2^x^	3.5944 (14)	Ni—Dy1^vi^	2.856 (2)
Dy2—Ni	2.740 (3)	Ni—Dy1^xiii^	3.3700 (15)
Dy2—Ni^xi^	2.790 (3)	Ni—Dy1^iii^	3.3700 (15)
Dy2—Dy1^v^	3.5361 (12)		
			
Ni^i^—Dy1—Ni	97.80 (5)	Dy1—Dy2—Dy1^vi^	60.06 (3)
Ni^i^—Dy1—Ni^ii^	110.14 (3)	Ni—Dy2—Dy1^viii^	111.46 (6)
Ni—Dy1—Ni^ii^	152.06 (4)	Ni^xi^—Dy2—Dy1^viii^	106.54 (6)
Ni^i^—Dy1—Dy1^iii^	132.42 (6)	Dy1^v^—Dy2—Dy1^viii^	59.11 (2)
Ni—Dy1—Dy1^iii^	62.83 (4)	Dy1^xii^—Dy2—Dy1^viii^	108.94 (3)
Ni^ii^—Dy1—Dy1^iii^	96.49 (5)	Dy1—Dy2—Dy1^viii^	59.67 (3)
Ni^i^—Dy1—Dy1^iv^	99.46 (5)	Dy1^vi^—Dy2—Dy1^viii^	89.35 (3)
Ni—Dy1—Dy1^iv^	127.71 (7)	Ni—Dy2—Dy1^i^	111.46 (6)
Ni^ii^—Dy1—Dy1^iv^	48.95 (4)	Ni^xi^—Dy2—Dy1^i^	106.54 (6)
Dy1^iii^—Dy1—Dy1^iv^	127.23 (4)	Dy1^v^—Dy2—Dy1^i^	108.94 (3)
Ni^i^—Dy1—Dy2^v^	110.13 (6)	Dy1^xii^—Dy2—Dy1^i^	59.11 (2)
Ni—Dy1—Dy2^v^	122.64 (5)	Dy1—Dy2—Dy1^i^	89.35 (3)
Ni^ii^—Dy1—Dy2^v^	47.57 (5)	Dy1^vi^—Dy2—Dy1^i^	59.67 (3)
Dy1^iii^—Dy1—Dy2^v^	61.27 (3)	Dy1^viii^—Dy2—Dy1^i^	59.12 (3)
Dy1^iv^—Dy1—Dy2^v^	96.43 (3)	Ni—Dy2—Dy1^xiii^	62.42 (3)
Ni^i^—Dy1—Dy2	73.05 (5)	Ni^xi^—Dy2—Dy1^xiii^	97.07 (4)
Ni—Dy1—Dy2	49.28 (6)	Dy1^v^—Dy2—Dy1^xiii^	155.48 (3)
Ni^ii^—Dy1—Dy2	139.07 (5)	Dy1^xii^—Dy2—Dy1^xiii^	59.64 (3)
Dy1^iii^—Dy1—Dy2	61.40 (2)	Dy1—Dy2—Dy1^xiii^	108.55 (3)
Dy1^iv^—Dy1—Dy2	170.23 (3)	Dy1^vi^—Dy2—Dy1^xiii^	58.947 (18)
Dy2^v^—Dy1—Dy2	92.13 (2)	Dy1^viii^—Dy2—Dy1^xiii^	144.99 (2)
Ni^i^—Dy1—Dy1^vi^	50.21 (4)	Dy1^i^—Dy2—Dy1^xiii^	89.83 (3)
Ni—Dy1—Dy1^vi^	51.63 (4)	Ni—Dy2—Dy1^iii^	62.42 (3)
Ni^ii^—Dy1—Dy1^vi^	153.03 (4)	Ni^xi^—Dy2—Dy1^iii^	97.07 (4)
Dy1^iii^—Dy1—Dy1^vi^	110.47 (2)	Dy1^v^—Dy2—Dy1^iii^	59.64 (3)
Dy1^iv^—Dy1—Dy1^vi^	110.47 (2)	Dy1^xii^—Dy2—Dy1^iii^	155.48 (3)
Dy2^v^—Dy1—Dy1^vi^	148.141 (19)	Dy1—Dy2—Dy1^iii^	58.947 (18)
Dy2—Dy1—Dy1^vi^	59.972 (15)	Dy1^vi^—Dy2—Dy1^iii^	108.55 (3)
Ni^i^—Dy1—Dy1^vii^	63.03 (5)	Dy1^viii^—Dy2—Dy1^iii^	89.83 (3)
Ni—Dy1—Dy1^vii^	160.83 (5)	Dy1^i^—Dy2—Dy1^iii^	144.99 (2)
Ni^ii^—Dy1—Dy1^vii^	47.11 (5)	Dy1^xiii^—Dy2—Dy1^iii^	112.85 (4)
Dy1^iii^—Dy1—Dy1^vii^	129.32 (4)	Ni—Dy2—Dy2^xiv^	48.35 (6)
Dy1^iv^—Dy1—Dy1^vii^	60.32 (3)	Ni^xi^—Dy2—Dy2^xiv^	87.67 (7)
Dy2^v^—Dy1—Dy1^vii^	68.17 (3)	Dy1^v^—Dy2—Dy2^xiv^	104.66 (3)
Dy2—Dy1—Dy1^vii^	119.32 (4)	Dy1^xii^—Dy2—Dy2^xiv^	104.66 (3)
Dy1^vi^—Dy1—Dy1^vii^	110.28 (2)	Dy1—Dy2—Dy2^xiv^	92.83 (3)
Ni^i^—Dy1—Dy1^viii^	51.95 (5)	Dy1^vi^—Dy2—Dy2^xiv^	92.83 (3)
Ni—Dy1—Dy1^viii^	109.78 (6)	Dy1^viii^—Dy2—Dy2^xiv^	146.39 (2)
Ni^ii^—Dy1—Dy1^viii^	88.29 (5)	Dy1^i^—Dy2—Dy2^xiv^	146.39 (2)
Dy1^iii^—Dy1—Dy1^viii^	91.96 (3)	Dy1^xiii^—Dy2—Dy2^xiv^	57.75 (2)
Dy1^iv^—Dy1—Dy1^viii^	119.71 (3)	Dy1^iii^—Dy2—Dy2^xiv^	57.75 (2)
Dy2^v^—Dy1—Dy1^viii^	61.14 (2)	Ni—Dy2—Dy2^xi^	176.75 (7)
Dy2—Dy1—Dy1^viii^	60.88 (2)	Ni^xi^—Dy2—Dy2^xi^	47.22 (6)
Dy1^vi^—Dy1—Dy1^viii^	90.0	Dy1^v^—Dy2—Dy2^xi^	59.55 (2)
Dy1^vii^—Dy1—Dy1^viii^	59.38 (3)	Dy1^xii^—Dy2—Dy2^xi^	59.55 (2)
Ni^i^—Dy1—Dy1^ix^	139.72 (4)	Dy1—Dy2—Dy2^xi^	125.27 (3)
Ni—Dy1—Dy1^ix^	49.80 (5)	Dy1^vi^—Dy2—Dy2^xi^	125.27 (3)
Ni^ii^—Dy1—Dy1^ix^	104.55 (5)	Dy1^viii^—Dy2—Dy2^xi^	65.79 (3)
Dy1^iii^—Dy1—Dy1^ix^	60.29 (3)	Dy1^i^—Dy2—Dy2^xi^	65.79 (3)
Dy1^iv^—Dy1—Dy1^ix^	88.04 (3)	Dy1^xiii^—Dy2—Dy2^xi^	118.64 (2)
Dy2^v^—Dy1—Dy1^ix^	108.20 (2)	Dy1^iii^—Dy2—Dy2^xi^	118.64 (2)
Dy2—Dy1—Dy1^ix^	93.77 (3)	Dy2^xiv^—Dy2—Dy2^xi^	134.90 (5)
Dy1^vi^—Dy1—Dy1^ix^	90.0	Dy2—Ni—Dy1^x^	140.04 (4)
Dy1^vii^—Dy1—Dy1^ix^	146.49 (3)	Dy2—Ni—Dy1^ix^	140.04 (4)
Dy1^viii^—Dy1—Dy1^ix^	150.16 (4)	Dy1^x^—Ni—Dy1^ix^	79.59 (8)
Ni^i^—Dy1—Dy2^x^	90.43 (6)	Dy2—Ni—Dy2^xiv^	84.43 (7)
Ni—Dy1—Dy2^x^	71.33 (6)	Dy1^x^—Ni—Dy2^xiv^	91.17 (8)
Ni^ii^—Dy1—Dy2^x^	107.50 (5)	Dy1^ix^—Ni—Dy2^xiv^	91.17 (8)
Dy1^iii^—Dy1—Dy2^x^	118.81 (4)	Dy2—Ni—Dy1	78.53 (7)
Dy1^iv^—Dy1—Dy2^x^	59.62 (2)	Dy1^x^—Ni—Dy1	126.22 (9)
Dy2^v^—Dy1—Dy2^x^	151.42 (2)	Dy1^ix^—Ni—Dy1	78.25 (5)
Dy2—Dy1—Dy2^x^	113.33 (3)	Dy2^xiv^—Ni—Dy1	137.35 (5)
Dy1^vi^—Dy1—Dy2^x^	60.442 (16)	Dy2—Ni—Dy1^vi^	78.53 (7)
Dy1^vii^—Dy1—Dy2^x^	106.94 (4)	Dy1^x^—Ni—Dy1^vi^	78.25 (5)
Dy1^viii^—Dy1—Dy2^x^	142.39 (2)	Dy1^ix^—Ni—Dy1^vi^	126.22 (9)
Dy1^ix^—Dy1—Dy2^x^	59.45 (3)	Dy2^xiv^—Ni—Dy1^vi^	137.35 (5)
Ni—Dy2—Ni3^xi^	136.02 (9)	Dy1—Ni—Dy1^vi^	76.74 (7)
Ni—Dy2—Dy1^v^	120.95 (2)	Dy2—Ni—Dy1^xiii^	71.46 (5)
Ni^xi^—Dy2—Dy1^v^	63.09 (3)	Dy1^x^—Ni—Dy1^xiii^	69.86 (3)
Ni—Dy2—Dy1^xii^	120.95 (2)	Dy1^ix^—Ni—Dy1^xiii^	142.80 (9)
Ni^xi^—Dy2—Dy1^xii^	63.09 (3)	Dy2^xiv^—Ni—Dy1^xiii^	69.34 (4)
Dy1^v^—Dy2—Dy1^xii^	116.28 (4)	Dy1—Ni—Dy1^xiii^	137.33 (9)
Ni—Dy2—Dy1	52.19 (4)	Dy1^vi^—Ni—Dy1^xiii^	68.22 (3)
Ni^xi^—Dy2—Dy1	149.959 (16)	Dy2—Ni—Dy1^iii^	71.46 (5)
Dy1^v^—Dy2—Dy1	87.87 (3)	Dy1^x^—Ni—Dy1^iii^	142.80 (9)
Dy1^xii^—Dy2—Dy1	144.38 (2)	Dy1^ix^—Ni—Dy1^iii^	69.86 (3)
Ni—Dy2—Dy1^vi^	52.19 (4)	Dy2^xiv^—Ni—Dy1^iii^	69.34 (4)
Ni^xi^—Dy2—Dy1^vi^	149.959 (16)	Dy1—Ni—Dy1^iii^	68.22 (3)
Dy1^v^—Dy2—Dy1^vi^	144.38 (2)	Dy1^vi^—Ni—Dy1^iii^	137.33 (9)
Dy1^xii^—Dy2—Dy1^vi^	87.87 (2)	Dy1^xiii^—Ni—Dy1^iii^	126.05 (8)

Symmetry codes: (i) *x*−1/2, −*y*+1/2, −*z*+1/2; (ii) −*x*+1/2, *y*−1/2, *z*−1/2; (iii) −*x*+1/2, −*y*, *z*+1/2; (iv) −*x*+1/2, −*y*, *z*−1/2; (v) −*x*, −*y*, −*z*+1; (vi) *x*, −*y*+1/2, *z*; (vii) −*x*, −*y*, −*z*; (viii) *x*−1/2, *y*, −*z*+1/2; (ix) *x*+1/2, *y*, −*z*+1/2; (x) *x*+1/2, −*y*+1/2, −*z*+1/2; (xi) *x*−1/2, −*y*+1/2, −*z*+3/2; (xii) −*x*, *y*+1/2, −*z*+1; (xiii) −*x*+1/2, *y*+1/2, *z*+1/2; (xiv) *x*+1/2, −*y*+1/2, −*z*+3/2.
